# Manganese-Functionalized Bentonite for Efficient Cadmium Ion Removal from Aqueous Systems

**DOI:** 10.3390/ma19112416

**Published:** 2026-06-05

**Authors:** Silvia Dolinská, Ingrid Znamenáčková, Věra Valovičová, Lenka Vaculíková, Slavomír Hredzák, Miroslava Václavíková, Lucia Ivaničová

**Affiliations:** 1 Institute of Geotechnics of the Slovak Academy of Sciences, 040 01 Košice, Slovakia; znamenackova@saske.sk (I.Z.); hredzak@saske.sk (S.H.); vaclavik@saske.sk (M.V.); ivanic@saske.sk (L.I.); 2 Institute of Geonics of the Czech Academy of Sciences, 708 00 Ostrava-Poruba, Czech Republic; vera.valovicova@ugn.cas.cz (V.V.); lenka.vaculikova@ugn.cas.cz (L.V.)

**Keywords:** bentonite, manganese oxide, X-ray photoelectron spectroscopy, composite materials, Cd^2+^ adsorption

## Abstract

**Highlights:**

- Bentonite modified to its sodium form and combined with quartz sand was treated with manganese oxides to prepare a low-cost sorbent.
- Characterization using XPS confirmed that manganese was predominantly present as Mn(IV) in MnO_2_-type phases.
- Birnessite-type manganese oxides form “sea urchin”-like spheres of interconnected nanowalls in a network structure.

**Abstract:**

Bentonite is widely used as a sorbent due to its high specific surface area and ion-exchange capacity; however, its properties can be significantly influenced by the presence of additional mineral phases and chemical modification. In this study, the influence of manganese oxides and quartz sand on the sorption properties of bentonite from the Stará Kremnička was systematically investigated, with particular attention to surface characterization by X-ray photoelectron spectroscopy (XPS). The materials were also characterized by X-ray diffraction, FTIR spectroscopy, and zeta potential measurements. XPS analysis revealed that manganese in all modified samples was predominantly present in the Mn(IV) oxidation state, with Mn 2p_3_/_2_ binding energies of 642.5–642.7 eV, corresponding to MnO_2_-type phases. Deconvolution of the O 1s spectra confirmed the presence of lattice oxygen, silicate oxygen, and surface hydroxyl groups. The reason for the modification of mainly natural materials with manganese oxides is their higher affinity for the adsorption of heavy metal cations. The maximum adsorption capacity of natural bentonite was 63.29 mg/g. In bentonite samples modified with manganese oxides, the value increased to 103.09 mg/g for BMn, and to 116.28 mg/g for the MMn mixture. The results demonstrate that sorption behavior is governed by a combination of ion exchange on bentonite and interactions with Mn oxide surface phases, providing new insight into the role of Mn(IV) species in surface-controlled metal binding processes. These findings highlight the importance of surface chemical states in designing efficient bentonite-based sorbents.

## 1. Introduction

Bentonite-based materials are widely used in sorption processes due to their ability to interact with dissolved metal ions through ion exchange and surface reactions [1,2,3,4,5]. These properties are primarily associated with montmorillonite, whose layered structure enables the exchange of interlayer cations and provides access to reactive surface sites [6,7,8,9,10,11]. However, in real systems, sorption behavior is not solely governed by the clay mineral itself but also by the presence of additional mineral phases and surface modification [3,12,13,14,15].

According to Ali et al., bentonite clay exhibited very high adsorption efficiency toward Pb(II) ions, with removal efficiencies exceeding 98% under optimized experimental conditions, demonstrating the strong affinity of bentonite surfaces toward dissolved heavy metals [16]. It was demonstrated that depending on the pH, ionic strength, composition of the solution and the conditions of the experiments, bentonites with different mineral composition and physical properties adsorb from 13.2 to 32.7 mg Cu^2+^ per gram clay. The main mechanisms of sorption of Cu(II) ions on bentonite are ion exchange and proton substitution of aluminol and silanol groups on the edge surfaces of clay minerals. Ion exchange is independent of pH [17,18,19].

Among potential modifiers, manganese oxides represent a group of materials with high surface reactivity and a strong affinity for metal ions [20,21,22]. Birnessite-type MnO_2_ is characterized by a layered structure composed of MnO_6_ octahedra with a high density of structural defects, such as vacancies or mixed Mn oxidation states, which lead to the formation of negatively charged surface sites. These features enable interactions with dissolved species through electrostatic attraction and surface complexation, making manganese oxides effective sorbents in environmental systems [21,23,24,25,26]. Previous studies have shown that modification of bentonite with manganese oxides can significantly enhance sorption capacity depending on the system composition and preparation method. At the same time, the practical application of manganese oxides alone is limited due to their fine particle size and poor handling properties, which has led to increasing interest in their immobilization on mineral supports [27,28].

During the modification process, native interlayer cations (e.g., Na^+^, Ca^2+^, K^+^) in montmorillonite can be partially exchanged with metal species present in the solution phase, including Mn-containing species. This ion-exchange process occurs through electrostatic interactions between hydrated Mn species and the negatively charged interlayer surfaces, resulting in partial displacement of the original exchangeable cations and/or the formation of inner-sphere or outer-sphere surface complexes [29,30]. In parallel, Mn oxide deposition on external clay surfaces may further modify interlayer spacing through steric constraints and surface charge alterations, which can lead to shifts in basal reflection positions observed in X-ray diffraction patterns [29]. The MnO_2_ particles formed via the redox precipitation reaction between KMnO_4_ and HCl are typically generated in the nanometer size range. According to literature reports for similar synthesis procedures, primary MnO_2_ particles commonly range from approximately 10 to 50 nm, although aggregation may lead to the formation of submicrometer clusters [31,32,33].

In addition, natural and engineered systems often contain mineral components that do not directly participate in sorption processes but may influence the overall system behavior. Quartz sand represents a typical example of a low-reactive mineral phase that can affect the spatial distribution of active components and the accessibility of surface sites without contributing significantly to chemical interactions. In such heterogeneous systems, interpreting sorption behavior requires consideration of both reactive and nonreactive components.

Although the sorption properties of manganese-modified bentonite have been reported in several studies [34,35], the relationship between surface chemical composition and sorption behavior remains insufficiently resolved. In particular, experimental studies often report enhanced sorption performance without establishing a direct correlation with specific surface chemical states, such as manganese oxidation states or the nature of surface oxygen species. As a result, the contribution of Mn(IV)-type phases and associated oxygen functionalities to sorption processes is often inferred rather than directly supported by surface-sensitive analysis.

However, despite numerous studies on manganese-modified bentonites, the relationship between specific surface chemical states—particularly manganese oxidation states and oxygen functionalities—and sorption performance remains insufficiently quantified, as enhanced sorption is often reported without direct correlation to surface-sensitive spectroscopic evidence. This limitation becomes more significant in heterogeneous systems containing additional mineral phases such as quartz sand, where the distribution and accessibility of surface-active components may vary considerably, while commonly used characterization methods provide only indirect or bulk information.

X-ray photoelectron spectroscopy (XPS) provides direct information about the elemental composition and chemical states within the uppermost few nanometers of a material surface [36,37,38]. In transition metal oxide systems, XPS enables the identification of oxidation states through the analysis of characteristic core-level spectra, such as Mn 2p, Fe 2p, Co 2p, and Ni 2p signals. In manganese oxides, Mn 2p spectra are commonly used to distinguish between Mn(II), Mn(III), and Mn(IV) species, while O 1s spectra provide additional information about lattice oxygen, hydroxyl groups, and adsorbed oxygen-containing species. Similar approaches are widely applied to iron, cobalt, and nickel oxides, where the complex multiplet splitting and satellite structures observed in Fe 2p, Co 2p, and Ni 2p spectra provide valuable insight into the chemical environment and oxidation states of surface species [37]. Previous studies have demonstrated that accurate interpretation of transition-metal XPS spectra requires careful consideration of multiplet splitting, shake-up satellites, and peak fitting procedures, particularly for mixed oxide and hydroxide systems. Therefore, the application of XPS to clay-based composite materials represents an important approach for correlating surface chemical properties with sorption behavior [38,39].

This study investigates the influence of manganese oxide modification and quartz sand on the sorption behavior of bentonite, with emphasis on linking surface chemical states identified by X-ray photoelectron spectroscopy (XPS) to sorption performance. Particular attention is given to manganese oxidation states and surface oxygen species, and their role in both single-phase and heterogeneous systems. This approach aims to clarify how Mn(IV)-related surface phases and oxygen functionalities govern sorption processes in composite systems.

## 2. Materials and Methods

**Figure 1:**
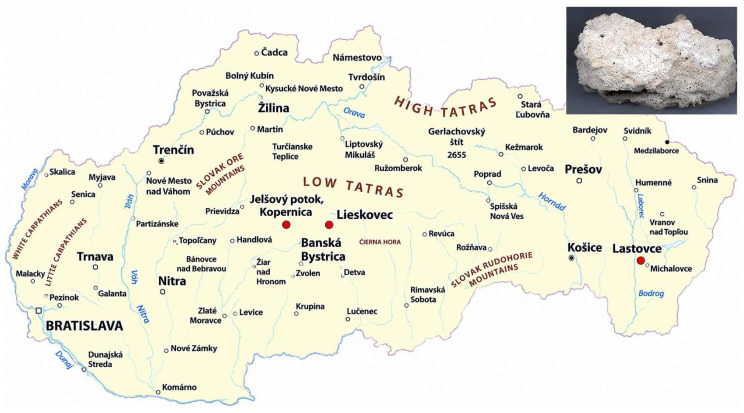
Natural Bentonite—locality Jelšový potok (map), Slovakia.

Natural bentonite came from the Stará Kremnička—Jelšový potok I deposit, Žiar nad Hronom district (Slovak Republic) Figure 1. The crystal chemical formula, according to Jesenák [40], can be expressed as:

[Si_7.95_Al_0.05_][Al_3.03_Fe_0.22_Mg_0.75_]·O_20_(OH)_4_·(Ca_0.42_Mg_0.04_Na_0.01_K_0.01_)

The Ca-montmorillonite content of mined bentonite ranges from 50 to 85%, followed by opal C/CT (5–25%), clinoptilolite (up to 15%), feldspars (3–12%), quartz (up to 8%), biotite (2–5%), and kaolinite (up to 2%). The bentonite sample used in this work was isolated from a 4% aqueous suspension of bentonite and processed by the sedimentation method in order to obtain an almost monomineralic fraction of montmorillonite (more than 90%) [40,41,42,43,44,45]. The particle size of starting bentonite material was under 20 µm. The first step in the chemical modification of natural bentonite was the preparation of the sodium form of bentonite. All chemicals and reagents used in this study were purchased from Mikrochem s.r.o. (Pezinok, Slovakia). The modification of selected bentonite samples (Table 1) with manganese oxides was carried out according to the procedure published by Sivasankar [46], as follows (1):

2KMnO_4_ + 8HCl → 2MnO_2_ + 2KCl + 3Cl_2_ + 4H_2_O (1)

Quartz sand came from the Šaštín-Stráže deposit (Slovak Republic). The extracted quartz sand was orange-brown in colour due to iron and clay minerals impregnated on the quartz grains. The quartz content was approximately 86%; the remainder was mainly feldspar, plagioclase, mica, kaolinite, and chlorite. Natural ferruginous quartz sand was obtained from a sampling point at a greater depth (around 0.5 m). According to the EN ISO 14688-1:2017, the particle size of the quartz sand ranged from 0.063 mm to 0.5 mm. The dominant fraction typically lies within the fine to medium sand range (approximately 0.125–0.5 mm) [47].

X-ray diffraction data were obtained using a Bruker D8 Advance diffractometer (Bruker, Karlsruhe, Germany) (40 kV, 40 mA) with Cu Kα radiation. The JCPDS (Joint Committee on Powder Diffraction Standards, International Center for Diffraction Data) database was used to analyze the diffraction peaks. The zeta potential of the studied samples was measured using a Zetasizer Nano-Z (Malvern Panalytical Ltd., Malvern, UK), operating on the principle of laser Doppler electrophoresis. The effect of pH on the zeta potential of particles in an aqueous environment was investigated. For each sample, a series of suspensions with a solid concentration of 2 g/L was prepared, and the pH was adjusted from 2 to 9 using NaOH and HNO_3_ solutions. The zeta potential measurements were performed immediately after pH adjustment of the suspensions. The infrared spectra were obtained by the KBr disc method on a Bruker Tensor 27 FTIR spectrometer (Bruker Optik GmbH, Ettlingen, Germany). For each sample, 64 scans were measured in the 4000–400 cm^−1^ spectral range in absorption mode at a resolution of 4 cm^−1^. X-ray photoelectron spectroscopy was carried out. X-ray photoelectron spectra (XPS) were obtained with a high-resolution electron spectrometer, VG ESCA 3 MK II (Gammadata Scienta, Uppsala, Sweden). The samples were spread onto gold plates, which were mounted on a sample probe using tantalum clips. Detailed spectral scans were taken over the Mn 2p, O 1s, C 1s, Si 2p, and K 2p spectral regions. The morphology and particle size of the samples were characterized using a field-emission scanning electron microscope (TESCAN MIRA 3 FE-SEM, Brno, Czech Republic) operated at 20 kV. The samples were examined at various magnifications to obtain detailed structural information. Batch adsorption experiments were performed to evaluate the sorption properties of the selected adsorbents. Cadmium uptake was investigated using synthetic Cd(NO_3_)_2_·4H_2_O solutions at temperature 25 °C. The initial Cd^2+^ concentrations ranged from 10 to 700 mg/L, while experiments examining the effects of contact time were conducted at a fixed concentration of 100 mg/L. The adsorbent dosage was maintained at 1 g/L. All experiments were carried out in polyethylene tubes placed on a rotary shaker at 150 rpm, and the solution pH was adjusted to 5 using NaOH and HNO_3_. The suspensions were agitated for 24 h to ensure equilibrium conditions.

Following equilibration, the residual Cd^2+^ concentration in the solution was determined by atomic absorption spectroscopy (AAS) using a Varian 240 RS/2400 instrument (Varian, Melbourne, Australia). The adsorption kinetics were analyzed using pseudo-second-order kinetic models (2):

(2) $$\frac{t}{{\;q}_{t}\;}=\frac{1}{k_{2}q_{e}^{2}}+\frac{t}{q_{e}}$$

where k_2_ (g·mg^−1^· min^−1^) is the rate constant of the pseudo-second-order model.

## 3. Results and Discussion

The main clay mineral in bentonite is montmorillonite, which makes bentonite a universal adsorbent for heavy metal cations due to its high adsorption capacity and cation exchange properties [8,48]. Other natural adsorbents include birnessite, a manganese oxide mineral that occurs naturally in soils and sediments and is characterized by high cation exchange capacity as well as strong sorption, oxidation, and catalytic properties [22]. Synthetically prepared manganese oxides of the birnessite type with a layered structure are as effective at capturing heavy metals as natural birnessite, while their preparation is relatively simple and fast and does not require many chemicals or expensive equipment. However, the produced oxides are present as a very fine powder, with particles of microscopic dimensions.

### 3.1. Phase Composition and Structural Analysis

**Figure 2:**
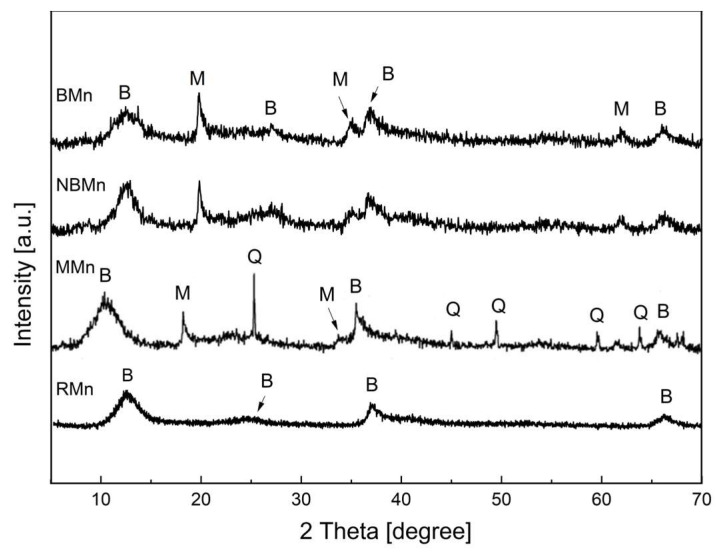
X-ray diffraction (XRD) patterns of reference manganese oxide (RMn), manganese-modified bentonite (BMn), manganese-modified sodium-activated bentonite (NBMn), and manganese-modified sodium-activated bentonite with quartz sand (MMn). M—montmorillonite; B—birnessite-type MnO_2_; Q—quartz.

X-ray diffraction (XRD) analysis showed that the natural bentonite is predominantly composed of montmorillonite, as indicated by the presence of its basal (001) reflection (d_001_ ≈ 15.14 Å) [41,42]. The reference manganese oxide sample exhibited diffraction features consistent with a birnessite-type structure, corresponding to a layered MnO_2_ phase with hexagonal symmetry. In the manganese-modified samples (BMn, NBMn, and MMn), noticeable changes in the diffraction patterns were observed. A decrease in the intensity of the montmorillonite (001) reflection and a reduction in higher-order reflections suggest partial coverage of the clay mineral surface and/or interaction between the layered silicate structure and the deposited manganese oxide phases. At the same time, additional reflections attributable to birnessite-type MnO_2_ confirm the presence of a separate manganese oxide phase within the composite system and support the deposition of manganese oxides onto the bentonite matrix. Quartz reflections were also identified in the composite samples (MMn), reflecting the presence of this inert mineral phase in the system (Figure 2).

The exchangeable cations (Na^+^, Ca^2+^, K^+^) originate from the natural structure of montmorillonite, which is the main mineral component of bentonite. These cations are located in the interlayer space and serve to balance the permanent negative charge of the clay layers, arising from isomorphic substitution within the octahedral and tetrahedral sheets.

During the modification process, these native interlayer cations can be partially exchanged with metal species present in the solution phase, including Mn-related species. An ion exchange process may occur when Mn species interact with the negatively charged interlayer surfaces, potentially leading to partial displacement of the original exchangeable cations. In parallel, Mn oxide deposition on external surfaces may further influence the interlayer spacing through steric effects and surface charge modification, resulting in the observed shifts in diffraction peak positions.

To further clarify the chemical state of manganese, the XPS measurements were applied. High-resolution spectra of the Mn 2p region were acquired over the binding energy range of 630–660 eV. The binding energies of Mn 2p and K 2p electrons, together with the full width at half maximum (FWHM) of the corresponding photoemission peaks (given in parentheses), are summarized in Table 2. The binding energies of C 1s and O 1s electrons, along with the FWHM values (in parentheses), are presented in Table 3.

**Figure 3:**
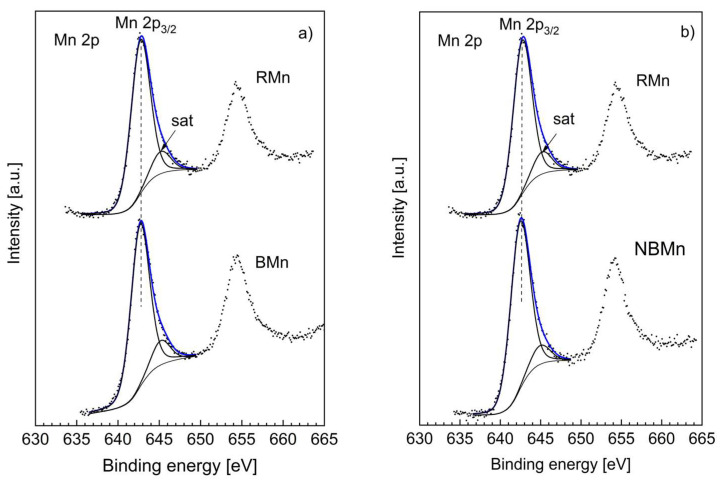
Comparison of photoelectron spectra Mn 2p3/2 samples: (**a**) reference manganese oxide (RMn), manganese-modified bentonite (BMn), (**b**) reference manganese oxide (RMn), manganese-modified sodium-activated bentonite (NBMn).

**Figure 4:**
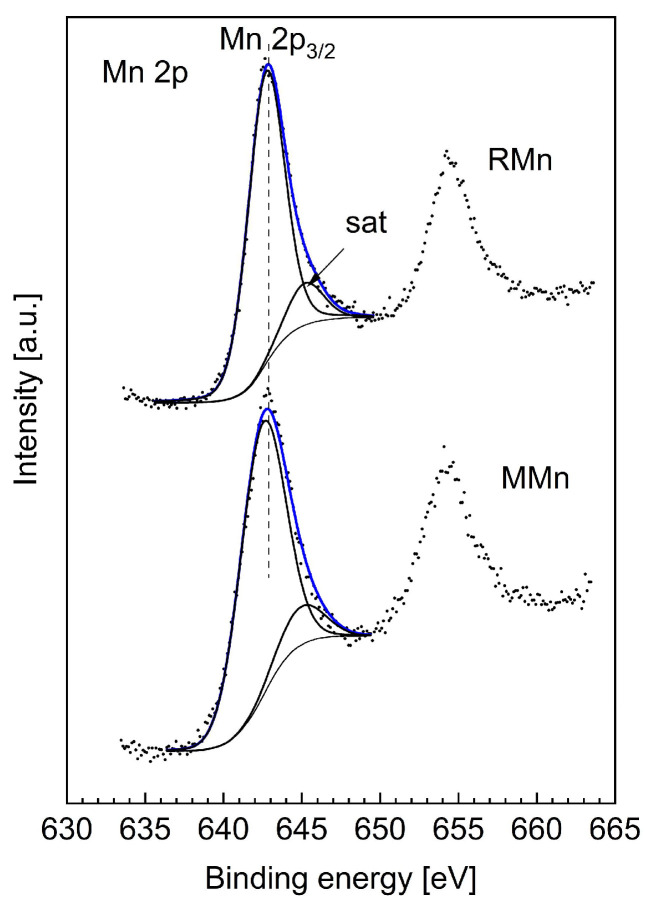
Comparison of photoelectron spectra Mn 2p_3/2_ samples: reference manganese oxide (RMn), manganese-modified sodium-activated bentonite with quartz sand (MMn).

The Mn 2p spectrum consists of a characteristic doublet: the main peak Mn 2p_3_/_2_ and its satellite Mn 2p_1_/_2_ components. The chemical state of manganese in all investigated samples was determined from the position of the Mn 2p_3_/_2_ peak. In the reference sample (RMn), a single chemical state corresponding to Mn^4+^ was identified, with a binding energy of 642.7 eV. Using the same approach, the Mn 2p_3_/_2_ spectra of BMn (Figure 3a), NBMn (Figure 3b), and MMn (Figure 4) were analyzed, revealing that manganese is present predominantly in the Mn^4+^ oxidation state in all samples. This result is consistent with the formation of birnessite-type MnO_2_ inferred from the XRD results.

**Figure 5:**
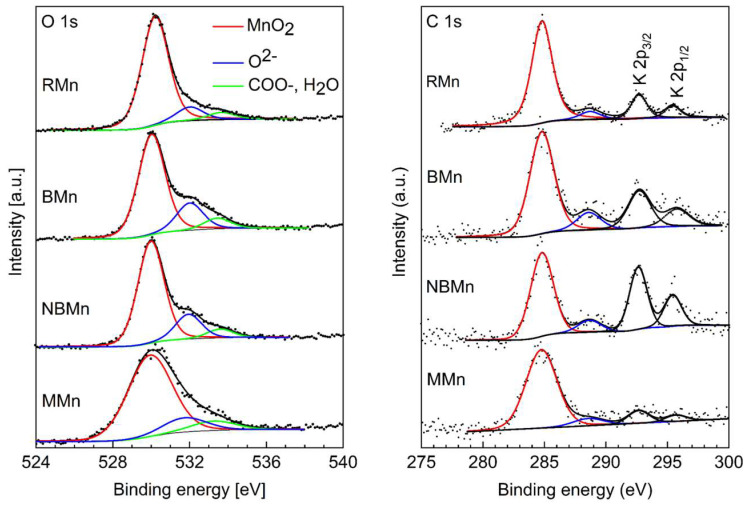
Photoelectron spectra O 1s and C 1s of samples: reference manganese oxide (RMn), manganese-modified bentonite (BMn), manganese-modified sodium-activated bentonite (NBMn), and manganese-modified sodium-activated bentonite with quartz sand (MMn).

Figure 5 shows the high-resolution O 1s and C 1s spectra for all manganese-containing samples. Deconvolution of the O 1s spectra indicates that oxygen is present in several chemical forms, including MnO_2_ (BE = 530.2 eV), oxides such as SiO_2_, K_2_O, MgO, and CaO (BE = 531.9 eV), and COO^−^ groups and adsorbed H_2_O (BE = 533.6 eV). In the C 1s region (Figure 5), in addition to carbon-related contributions, the K 2p doublet (K 2p_3_/_2_ and K 2p_1_/_2_) was also identified.

**Figure 6:**
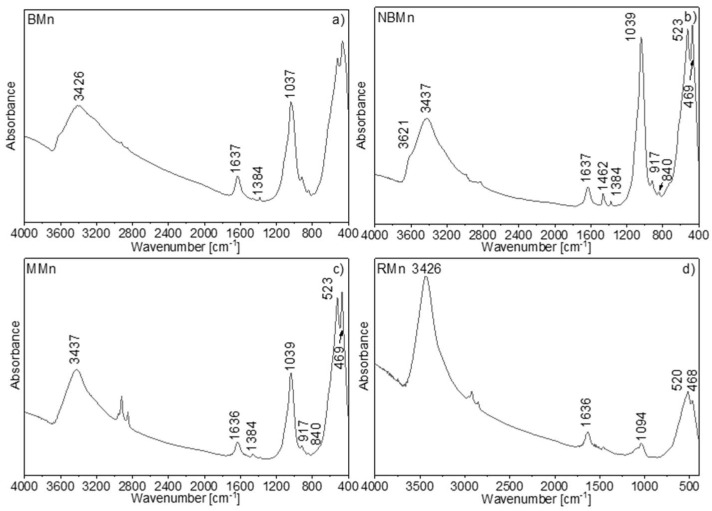
FTIR spectra of (**a**) manganese-modified bentonite (BMn), (**b**) manganese-modified sodium-activated bentonite (NBMn), (**c**) manganese-modified sodium-activated bentonite with quartz sand (MMn) and (**d**) reference manganese oxide (RMn).

Fourier transform infrared (FTIR) spectroscopy further supports structural changes induced by manganese oxide modification. Based on previously published results, the broad band at 3426 cm^−1^ corresponds to O-H stretching vibrations of structural hydroxyl groups in montmorillonite, while the band at 1637 cm^−1^ is associated with bending vibrations of adsorbed water molecules [41,42]. In the NBMn sample, the O-H stretching band shifted to higher wavenumbers (3437 cm^−1^), which may be related to changes in the hydration environment induced by sodium activation. The bands at 917 and 841 cm^−1^ correspond to Al–Al–OH and Al–Mg–OH bending vibrations, while the band at 1039 cm^−1^ is assigned to Si–O stretching vibrations of the aluminosilicate framework. In Mn-modified samples (BMn, NBMn, and MMn), additional bands at 1637 and 1384 cm^−1^ were observed, related to water-associated O-H vibrations influenced by manganese oxide deposition. The FTIR spectrum of the reference manganese oxide sample (RMn) does not fully correspond to standard MnO_2_ spectra reported in the literature [49]. However, weak bands at 520 and 468 cm^−1^ can be attributed to O–Mn–O bending vibrations. In the FTIR spectrum of RMn, bands at 3426 cm^−1^ and 1636 cm^−1^ were also confirmed, which belong to the O-H group vibrations of absorbed water molecules. In Mn-modified bentonite samples (NBMn and MMn), the identification of MnO_2_-related bands remains inconclusive due to strong overlap with Si–O vibrations of the clay mineral (≈523 and 469 cm^−1^), which limits direct spectroscopic discrimination of manganese oxide phases (Figure 6).

Importantly, the combined XRD, XPS, and FTIR results provide complementary information on the structure and surface chemistry of the investigated systems. While XRD confirms the presence of crystalline birnessite-type MnO_2_ phases and their interaction with montmorillonite, XPS demonstrates that manganese remains predominantly in the Mn(IV) oxidation state across all samples. FTIR, although limited by spectral overlap, supports changes in the hydroxyl environment and water coordination induced by manganese oxide deposition.

In addition, BET analysis revealed differences in the specific surface area and pore structure of the investigated materials. The obtained SBET values indicate that manganese oxide deposition influenced the surface properties and porosity of the montmorillonite-based systems. The specific surface area was calculated from the adsorption isotherms following the BET (Brunauer–Emmett–Teller) method within the relative pressure range of 0.05–0.3 p/p0. The measured value of surface properties, namely BET surface area (S_BET_), micropore volume (V_mikro_) and external surface area (S_t_), are summarized in Table 4.

The higher specific surface area was found for the NBMn sample. The specific surface area of the reference manganese oxide sample (RMn) correlates with the specific surface area (Table 4) for a synthetic surface prepared according to the McKenzie method, which was published by Cheney [50]. Precipitation of manganese oxides on the surface of natural bentonite caused its reduction. The specific surface area of MMn was comparable to that of BMn.

**Figure 7:**
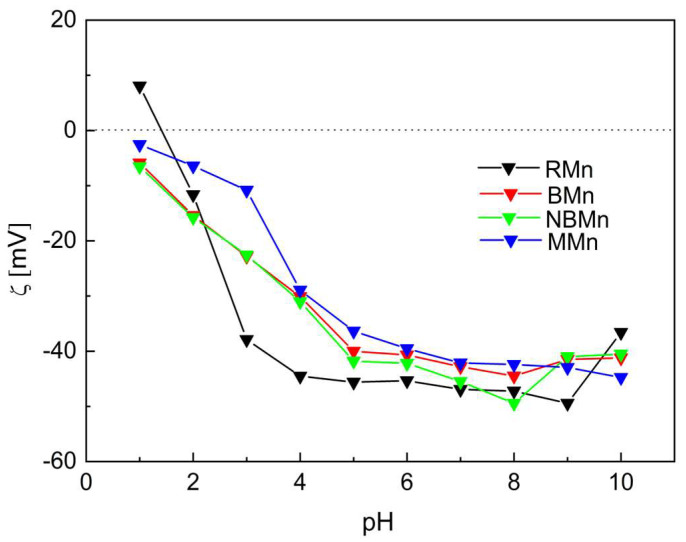
Zeta potential as a function of pH for reference manganese oxide (RMn), manganese-modified bentonite (BMn), manganese-modified sodium-activated bentonite (NBMn), and manganese-modified sodium-activated bentonite with quartz sand (MMn).

The reference manganese oxide sample with a birnessite-type structure is composed of layers of Mn^4+^O_6_ octahedra, where structural defects such as vacancies or substitution of Mn^4+^ by Mn^3+^ frequently occur. These defects in the crystal lattice result in a negative charge of the birnessite structure, which is compensated by various hydrated cations (mainly Ca, Na, K, etc.) located in the interlayer space of the manganese oxide structure. However, the exchange of individual cations during Cd^2+^ adsorption was not directly monitored in the present work. Figure 7 shows the dependence of the zeta potential of the RMn sample on pH in an aqueous environment. Starting from positive values at pH = 1, the zeta potential passes through an isoelectric point around pH ≈ 1.5 and then shifts to negative values, remaining stable (absolute value higher than 30 mV) over a wide pH range (from 3 to 10). The precipitation of birnessite-type manganese oxides onto the surface of natural and sodium-activated bentonite (BMn and NBMn) resulted in more negative zeta potential values compared to the previously characterized unmodified bentonite system. These results suggest that, due to stronger repulsive interactions between BMn and NBMn particles, the modified samples exhibit improved stability in aqueous suspension relative to the previously characterized unmodified bentonite materials [42]. The zeta potential of the mixture of sodium-activated bentonite and quartz sand modified with manganese oxides (MMn) was negative over the entire pH range, with values exceeding −30 mV already at pH = 4 (Figure 7).

### 3.2. SEM–EDX Characterization of Prepared Materials

**Figure 8:**
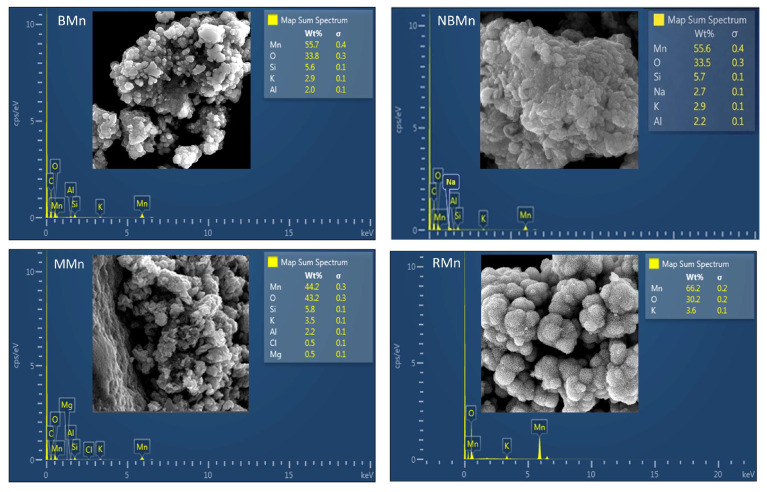
SEM images with EDX analysis of reference manganese oxide (RMn), manganese-modified bentonite (BMn), manganese-modified sodium-activated bentonite (NBMn), and manganese-modified sodium-activated bentonite with quartz sand (MMn).

The morphology of the prepared birnessite-type manganese oxides (RMn) is shown in Figure 8. The material exhibited a characteristic “sea urchin”-like morphology, composed of thin oxide sheets (nanowalls) with thicknesses on the order of several tens of nanometers, consistent with the morphology reported for birnessite-type MnO_2_ by Zhu et al. [51]. These nanowalls are interconnected and randomly oriented, extending toward the center and arranged perpendicular to the sample surface, thus forming a network structure. This network was subsequently assembled into an energetically favorable spherical morphology (Figure 8). In this form, it either constituted aggregates of individual oxides (RMn) or was incorporated into modified samples of natural bentonite (BMn), sodium-activated bentonite (NBMn), and a mixture of sodium-activated bentonite with quartz sand (MMn). These observations are consistent with the XRD results, which indicate the presence of manganese oxide phases in the composite.

The elemental composition of the reference manganese oxide sample (RMn) confirmed the presence of Mn, K, and O (Figure 8), in agreement with the expected composition resulting from the reductive precipitation of manganese dioxide (Reaction 1). In the case of BMn, Si, Al, Mn, O, and K were detected, whereas in NBMn, Si, Al, Mn, O, K, and Na were identified (Figure 8). Sodium detected in NBMn reflects the sodium activation of the bentonite precursor. The presence of potassium can be attributed to residual species originating from the precipitation process. In the case of MMn, the local EDX analysis showed a mixed elemental composition corresponding to the coexistence of manganese oxide and silicate components within the composite.

The surface properties described above are reflected in the sorption of the studied materials. The more negative surface charge observed for BMn and NBMn over a wide pH range indicates an increased number of negatively charged surface sites, which can promote the adsorption of Cd^2+^ ions. This is consistent with their higher stability in suspension and suggests improved availability of active sites compared to the unmodified samples. In the case of MMn, the zeta potential remained negative over the entire pH range, with values exceeding −30 mV already at pH 4. This indicates a stable dispersion and a significant contribution of manganese oxide phases to the surface charge. However, the presence of quartz, which is largely inert, likely affects the spatial distribution and accessibility of active sites rather than their intrinsic reactivity, thereby influencing the overall sorption performance of this system.

### 3.3. Adsorption Behavior

#### Adsorption Kinetics of Cd (II)

The sorption behavior of Cd^2+^ on the studied materials was evaluated under controlled conditions, with pH 5 selected to minimize precipitation effects and ensure that the observed removal was predominantly governed by adsorption [52]. The pH value reported (pH = 5) corresponds to the experimental conditions, which were continuously monitored and maintained throughout the sorption experiments. This pH range is consistent with previously reported studies on bentonite-based sorbents, where stable Cd^2+^ adsorption has been observed under mildly acidic conditions [41,42,53]. Under these conditions, differences between individual samples can be attributed primarily to variations in surface properties rather than to changes in Cd speciation.

**Figure 9:**
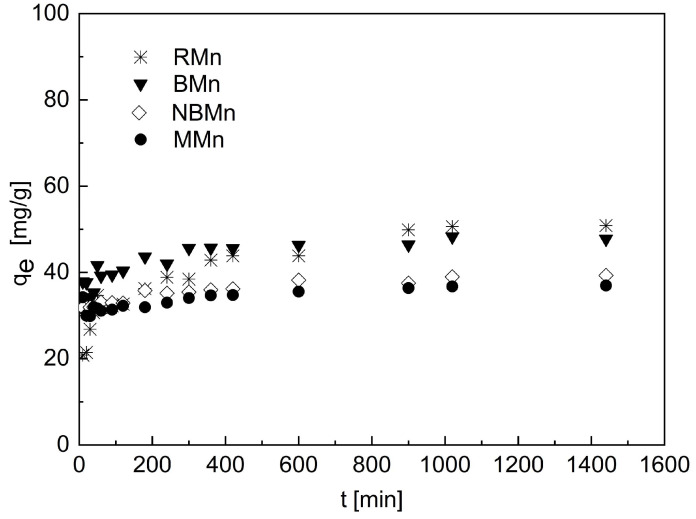
Dependence of the adsorbed amount of Cd^2+^ on the sorption time for reference manganese oxide (RMn), manganese-modified bentonite (BMn), manganese-modified sodium-activated bentonite (NBMn), and manganese-modified sodium-activated bentonite with quartz sand (MMn).

The time-dependent adsorption data (Figure 9) indicate a gradual increase in the amount of adsorbed Cd^2+^ for all manganese-modified samples (BMn, NBMn, MMn), approaching equilibrium after approximately 16 h, with only minor changes at longer contact times.

The experimentally observed sorption efficiency after 24 h followed the order MMn < NBMn < RMn ≈ BMn, corresponding to removal efficiencies of 39.21% for MMn, 70.84% for NBMn, 74.20% for RMn, and 75.16% for BMn. This trend reflects differences in the availability and accessibility of active MnO_2_-related surface sites.

**Figure 10:**
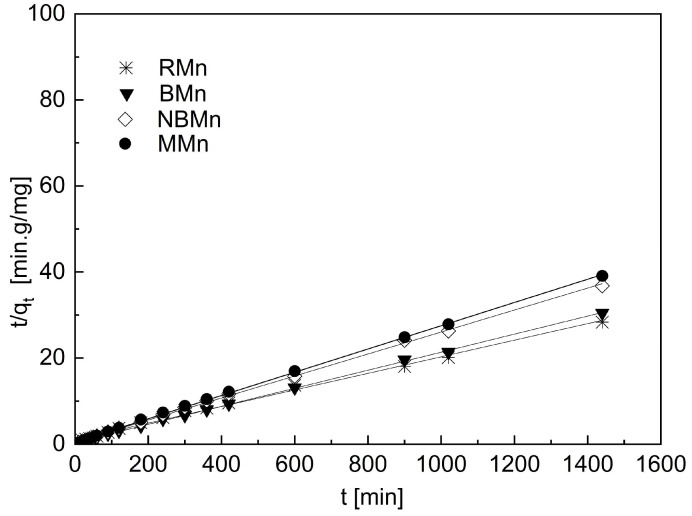
Pseudo-second order kinetic model for Cd^2+^ removal for samples: reference manganese oxide (RMn), manganese-modified bentonite (BMn), manganese-modified sodium-activated bentonite (NBMn), and manganese-modified sodium-activated bentonite with quartz sand (MMn).

The agreement with the pseudo-second-order kinetic model (2) (Figure 10) suggests that the sorption process is predominantly associated with surface-related interactions, although contributions from diffusion cannot be excluded [54,55].

Table 5 shows the parameters for the second-order kinetic model, observing a correlation between the experimental (qe (exp)) and theoretical (qe (cal)) values of the adsorbed amount of Cd^2+^, confirming that this model was appropriately used for all studied samples.

**Table 5:** Parameters of the pseudo-second-order kinetic model for Cd^2+^ sorption on the studied materials.

Sample	q_e_ (exp) [mg/g]	q_e_ (cal) [mg/g]	k_2_ [g/mol·min]	R^2^
BMn	47.36	47.62	0.0013	0.9994
NBMn	39.22	39.21	0.0014	0.9991
MMn	36.90	37.03	0.0017	0.9994
RMn	50.84	51.81	0.0003	0.9947

Although Mn(IV) species were identified in all manganese-modified samples, the relatively higher sorption performance of BMn and RMn is likely related to differences in the accessibility and distribution of reactive manganese oxide surface sites. The predominance of Mn(IV) species is consistent with the presence of birnessite-type MnO_2_ phases in all studied samples. Such Mn(IV)-dominated phases are characterized by a high density of negatively charged surface sites associated with structural defects and oxygen-containing functional groups. The O 1s spectra are consistent with contributions from lattice oxygen, silicate oxygen, and surface hydroxyl groups, which may represent potential surface sites for Cd^2+^ binding, consistent with adsorption mechanisms previously reported for MnO_2_-containing systems [56,57]. These results suggest that sorption in manganese-modified bentonite systems reflects the combined contribution of the bentonite matrix, including the known cation-exchange properties of bentonite, and interactions with manganese oxide surface phases.

The lower sorption efficiency of NBMn, despite its more negative zeta potential, indicates that electrostatic attraction alone does not control Cd^2+^ uptake. The more negative surface charge may facilitate the initial approach of Cd^2+^ ions to the surface; however, the overall sorption capacity appears to depend primarily on the accessibility and distribution of reactive MnO_2_-related sites. Sodium activation may also influence the structural arrangement of the bentonite matrix, thereby affecting the accessibility of these active sites.

The lowest sorption efficiency observed for MMn can be attributed to the presence of quartz sand as a non-reactive component. Although XPS confirms the presence of Mn(IV) species in this sample, the dilution of active phases and their heterogeneous spatial distribution likely reduce the effective contact between Cd^2+^ ions and reactive surface sites. In this context, quartz sand does not contribute significantly to sorption, but it affects the organization of the composite system and thereby influences the overall sorption efficiency.

**Figure 11:**
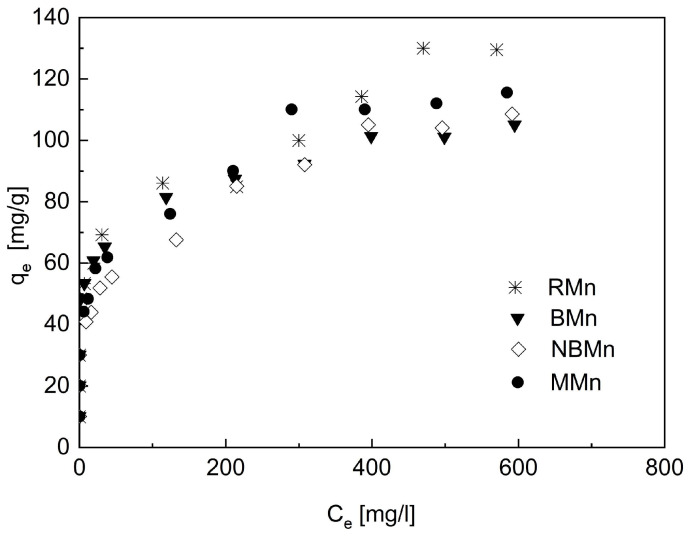
Dependence of the adsorbed amount of Cd^2+^ on its concentration for samples: reference manganese oxide (RMn), manganese-modified bentonite (BMn), manganese-modified sodium-activated bentonite (NBMn), and manganese-modified sodium-activated bentonite with quartz sand (MMn).

The adsorbed amount of Cd^2+^ increased continuously with increasing initial concentration in the range 10–700 mg/L (Figure 11), showing no distinct plateau within the investigated range. Such behavior is consistent with heterogeneous sorption systems under limited concentration ranges.

**Figure 12:**
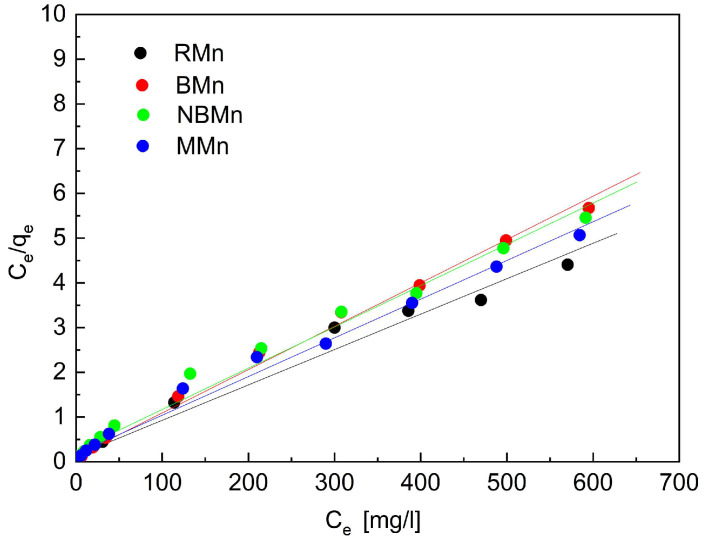
Langmuir isotherm for Cd^2+^ sorption for samples: reference manganese oxide (RMn), manganese-modified bentonite (BMn), manganese-modified sodium-activated bentonite (NBMn), and manganese-modified sodium-activated bentonite with quartz sand (MMn).

**Figure 13:**
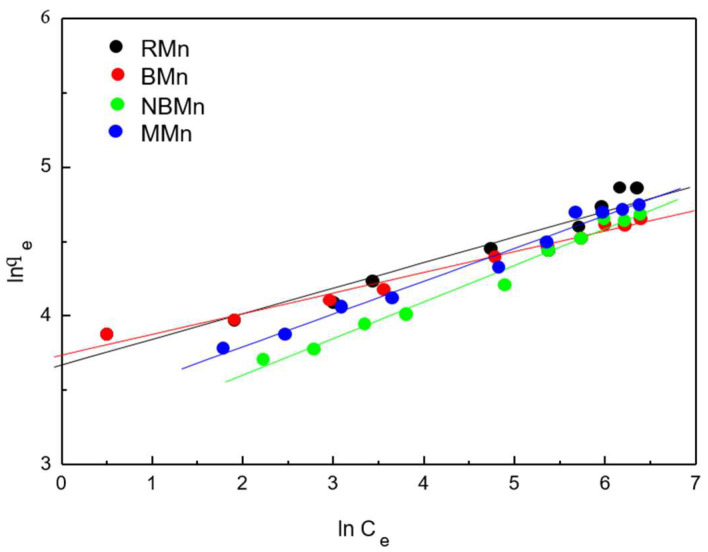
Freundlich isotherm for Cd^2+^ sorption for samples: reference manganese oxide (RMn), manganese-modified bentonite (BMn), manganese-modified sodium-activated bentonite (NBMn), and manganese-modified sodium-activated bentonite with quartz sand (MMn).

The sorption data were evaluated using both Langmuir and Freundlich isotherm models. For most samples, the Langmuir model provided a slightly better description of the experimental data (Figure 12, Table 6), although the Freundlich model also showed reasonably good agreement (Figure 13, Table 6).

**Table 6:** Langmuir and Freundlich adsorption constants for Cd^2+^ sorption on the studied materials.

Sample	Q_m_ [mg/g]	K [L/mg]	R^2^ Langmuir	1/*n*	KF [mg^−1−1/n^L^1/n^ g^−1^]	R^2^ Freundlich
BMn	103.09	0.083	0.9948	0.139	41.971	0.9809
NBMn	108.69	0.038	0.9847	0.246	22.443	0.9841
MMn	116.28	0.050	0.9895	0.220	28.674	0.9815
RMn	126.58	0.058	0.9826	0.172	39.330	0.9294

The maximum adsorption capacities calculated from the Langmuir model were significantly higher for the modified samples, reaching 103.09 mg/g for BMn, 108.69 mg/g for NBMn, 116.28 mg/g for MMn, and 126.58 mg/g for RMn, compared to 63.29 mg/g for unmodified natural bentonite. The calculated Qm values obtained from Langmuir isotherm fitting reflect the adsorption behavior over the investigated concentration range and therefore do not directly correspond to the adsorption efficiencies observed in the kinetic experiments.

Although the reference manganese oxide (RMn) exhibited high sorption efficiency, its practical application is limited by its fine particle size. In this respect, manganese-modified bentonite materials represent a more suitable alternative, combining effective sorption behavior with improved structural stability and handling. Despite moderate differences in the calculated Langmuir capacities, all manganese-modified materials exhibited relatively high Cd^2+^ adsorption performance under the investigated conditions, suggesting their potential applicability as low-cost sorbents for aqueous systems. Potential practical limitations include swelling behaviour and difficulties in solid–liquid separation associated with suspension formation. Overall, the results suggest that Cd^2+^ sorption in the studied systems reflects the combined contribution of the bentonite component, including the known cation-exchange properties of bentonite, and interactions with manganese oxide surface phases, while XPS provides direct information on the surface chemical states relevant to this behavior.

## 4. Conclusions

The results of this study demonstrate that the modification of bentonite with manganese oxide leads to the formation of MnO_2_-type surface phases, in which manganese is predominantly present in the Mn(IV) oxidation state, as confirmed by X-ray photoelectron spectroscopy (XPS). The prepared materials (BMn, NBMn, MMn) exhibit distinct sorption behaviors depending on the accessibility and distribution of manganese oxide phases. The Langmuir model yielded a calculated maximum adsorption capacity of 116.28 mg/g for MMn, although the presence of quartz sand likely reduced the accessibility of active sorption sites. From an economic standpoint, the incorporation of quartz sand into bentonite-based mixtures is advantageous, as it allows partial substitution of the active component with a low-cost and readily available material without a proportional loss in sorption efficiency. The sorption of Cd^2+^ is interpreted as reflecting the combined contribution of the bentonite component, including its ion-exchange properties, and interactions with manganese oxide surface phases. The kinetic data are well described by the pseudo-second-order model, suggesting that surface-related processes play a dominant role. The adsorption isotherms were fitted using both the Langmuir and Freundlich models; however, no distinct saturation plateau was observed within the investigated concentration range, which is consistent with the heterogeneous nature of the studied systems. The results indicate that variations in surface chemical composition and structure are directly reflected in sorption behavior and confirm that XPS is a suitable tool for correlating surface chemical states with adsorption performance in complex multicomponent systems. These findings provide a basis for the development of cost-effective sorbents for the removal of heavy metals from aqueous media.

## Figures and Tables

**Table 1:** Modified bentonite samples and reference material.

Samples	
BMn: manganese-modified bentonite	natural bentonite modified with manganese oxides in a ratio (bentonite: MnO_2_ = 1:1)
NBMn: manganese-modified sodium-activated bentonite	natrified bentonite modified with manganese oxides in a ratio (natrified bentonite: MnO_2_ = 1:1)
MMn: manganese-modified sodium-activated bentonite with quartz sand	mixture of natrified bentonite and natural quartz sand, mechanically homogenized in a ratio (1:1), modified with manganese oxides in a ratio (mixture: MnO_2_ = 1:1)
RMn: reference manganese oxide	in order to compare the structural and adsorption properties of oxides and modified bentonites, a fine powder reference sample of manganese oxides was prepared by the precipitation method (1)

**Table 2:** Binding energies of inner-shell electrons and widths of the photoemission lines at half height for potassium and manganese.

Samples	K (2p_3/2_)	Mn (2p_3/2_)	
	K^+^	Mn^4+^	Sat.
RMn	292.7 (1.42)	642.7 (2.75)	645.2 (2.75)
BMn	292.7 (2.10)	642.7 (2.77)	645.2 (2.77)
NBMn	292.6 (1.68)	642.5 (2.82)	645.0 (2.82)
MMn	292.7 (2.95)	642.5 (3.39)	645.0 (3.39)

All values are in eV. Widths of the photoemission lines at half-height (in parentheses).

**Table 3:** Binding energies of inner-shell electrons and widths of the photoemission lines at half-height for carbon and oxygen.

Samples	C 1s		O 1s		
	C-C	COO-	MnO_2_	COO-, H_2_O	O^2−^
RMn	284.8 (1.99)	288.7 (1.99)	530.2 (1.64)	533.7 (1.64)	532.0 (1.64)
BMn	284.8 (2.30)	288.6 (2.30)	530.0 (1.64)	533.4 (1.64)	532.0 (1.64)
NBMn	284.8 (2.18)	288.7 (2.18)	530.0 (1.60)	533.6 (1.60)	531.9 (1.60)
MMn	284.8 (2.95)	288.6 (2.95)	530.2 (2.70)	533.3 (2.70)	531.9 (2.70)

All values are in eV. Widths of the photoemission lines at half-height (in parentheses).

**Table 4:** Surface and porous parameters of studied samples.

Sample	S_BET_ [m^2^/g]	V_mikro_ [cm^3^/g]	S_t_ [m^2^/g]
BMn	29.8	0.002	26.7
NBMn	68.4	0.006	56.3
MMn	29.3	0.002	25.9
RMn	39.6	0	40.3

## Data Availability

The original contributions presented in this study are included in the article. Further inquiries can be directed to the corresponding author.
